# The Value of a Coronary Computed Tomography Angiography plus Stress Cardiac Magnetic Resonance Imaging Strategy for the Evaluation of Patients with Chronic Coronary Syndrome

**DOI:** 10.3390/jcm13061556

**Published:** 2024-03-08

**Authors:** Gherardo Busi, Mattia Alexis Amico, Matteo Vannini, Giacomo Virgili, Angela Migliorini, Giulia Pontecorboli, Silvia Pradella, Manlio Acquafresca, Mario Moroni, Carlo Di Mario, Renato Valenti, Nazario Carrabba

**Affiliations:** 1 Cardio-Thoraco-Vascular Department, Careggi Hospital, 50134 Florence, Italy; gherardobusi@gmail.com (G.B.); mattiaalexis.amico@unifi.it (M.A.A.); matteovannini2@me.com (M.V.); virgio9.gv@gmail.com (G.V.); migliorinia@aou-careggi.toscana.it (A.M.); pontecorbolig@aou-careggi.toscana.it (G.P.); carlo.dimario@unifi.it (C.D.M.); valentir@aou-careggi.toscana.it (R.V.); 2 Department of Radiology, Careggi Hospital, 50134 Florence, Italy; pradellas@aou-careggi.toscana.it (S.P.); acquafrescam@aou-careggi.toscana.it (M.A.); elleci77@libero.it (M.M.)

**Keywords:** coronary computed tomography angiography, stress cardiac magnetic resonance imaging, chronic coronary syndrome, intermediate coronary plaques, guideline-directed medical therapy, revascularization

## Abstract

**Background:** Noninvasive imaging methods, either anatomical or functional tests, serve as essential instruments for the appropriate management of patients with established or suspected coronary artery disease (CAD). We sought to evaluate the safety and efficacy of a coronary computed tomography angiography (CCTA) plus stress cardiac magnetic resonance imaging (S-CMR) strategy in patients with chronic coronary syndrome (CCS). **Methods:** Patients with suspected CCS showing intermediate coronary plaques (stenosis 30–70%) at CCTA underwent S-CMR. Patients with a positive S-CMR were referred to invasive coronary angiography (ICA) plus instantaneous wave-free ratio (iFR), and myocardial revascularization if recommended. All patients received guideline-directed medical therapy (GDMT), including high-dose statins, regardless of myocardial revascularization. The primary endpoint was a composite of death from cardiovascular causes, non-fatal myocardial infarction, and unplanned revascularization. **Results:** According to the results of CCTA, 62 patients showing intermediate coronary plaques underwent S-CMR, which was positive for a myocardial perfusion deficit in *n* = 17 (27%) and negative in *n* = 45 (73%) patients. According to the results of ICA plus iFR, revascularization was performed in 13 patients. No differences in the primary endpoint between the positive and negative S-CMR groups were observed at 1 year (1 [5.9%] vs. 1 [2.2%], *p* = 0.485) and after a median of 33.4 months (2 [11.8%] vs. 3 [6.7%]; *p* = 0.605). **Conclusions:** Our study suggests that a CCTA plus S-CMR strategy is effective for the evaluation of patients with suspicion of CCS at low–intermediate risk, and it may help to refine the selection of patients with intermediate coronary plaques at CCTA needing coronary revascularization.

## 1. Introduction

Coronary artery disease (CAD) is the leading cause of death and disability worldwide, leading to a high burden of heart failure and mortality, with 17.8 million deaths annually [1]. Approximately 1 in 30 patients with stable CAD experiences cardiovascular death or myocardial infarction every year [2]. Novel changes in the diagnostic workup of suspected obstructive CAD (O-CAD) were introduced in the European Society of Cardiology (ESC) guidelines on the diagnosis and management of chronic coronary syndromes (CCSs) [3]. Noninvasive imaging methods, either anatomical or functional tests, serve as essential instruments for the appropriate management of patients with established or suspected CAD: they provide reliable detection of the disease, help guide therapy, and contribute to outcome prediction. For this purpose, European and Italian guidelines recommend the use of coronary computed tomography angiography (CCTA) or stress imaging tests for the evaluation of patients with CCS to select those who would benefit from a more targeted management [3,4]. However, in patients with CCS, the current clinical practice does not adopt guidelines recommendations on the use of diagnostic tests in a significant proportion of patients. Importantly, when the diagnostic approach adopts guideline recommendations, invasive procedures are less frequently used, and the diagnostic yield and therapeutic utility are superior [5].

In clinical practice, in case of intermediate coronary plaques, most patients undergo invasive coronary angiography (ICA) and revascularization. Stress cardiac magnetic resonance imaging (S-CMR) is a functional imaging test that in recent years has been extensively acknowledged as an accurate, thoroughly validated, and non-ionizing technique [6,7,8]. Recently, the MR INFORM trial demonstrated that S-CMR is non-inferior to ICA and measurement of fractional flow reserve (FFR) in guiding the management of patients with stable CAD with respect to major adverse cardiac events, and it is associated with a lower incidence of coronary revascularization [9]. To assess if a S-CMR-based approach performed well also in patients with intermediate coronary plaques, the purpose of our study was to evaluate the safety and efficacy of a CCTA plus S-CMR strategy in patients with CCS.

## 2. Materials and Methods

**Figure 1:**
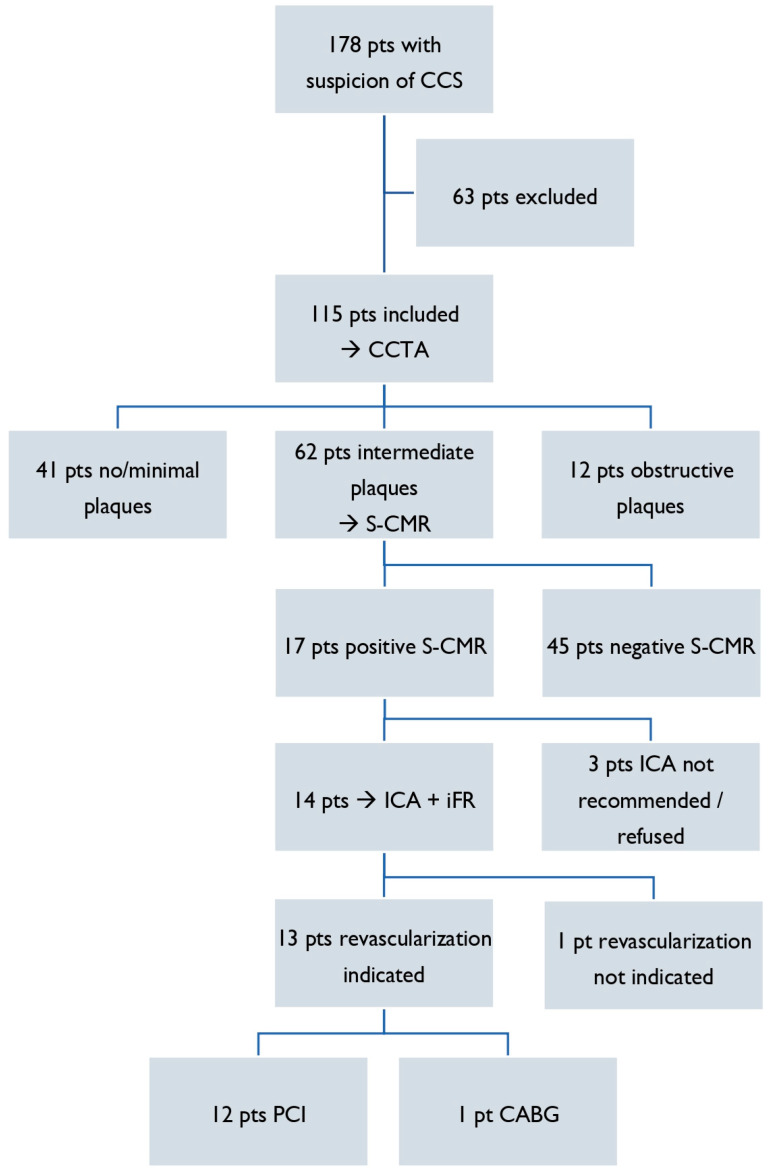
Flow diagram of study population. CABG, coronary artery bypass grafting; CCS, chronic coronary syndrome; CCTA, coronary computed tomography angiography; ICA, invasive coronary angiography; iFR, instantaneous wave-free ratio; PCI, percutaneous coronary intervention; pts, patients; S-CMR, stress cardiac magnetic resonance imaging.

From January 2019 to December 2021, 178 consecutive outpatients ≥18 years old with suspicion of CCS were referred to our Cardio-Thoraco-Vascular Department at Careggi Hospital (Florence, Italy) (see flow diagram, Figure 1).

Exclusion criteria were acute coronary syndrome, clinical instability or recent (<3 month) myocardial infarction (*n* = 41), contraindications to CCTA (*n* = 6), contraindication to S-CMR (*n* = 11, cerebral clips, eye metallic clips, known allergy to gadolinium-based contrast medium, estimated glomerular filtration rate < 30 mL/min/1.73 m^2^), need for emergent or urgent procedure (*n* = 1), and recent (<90 days) cardiovascular testing (*n* = 4). Importantly, despite recent radiologic guidelines no longer supporting an estimated glomerular filtration rate <30 mL/min/1.73 m^2^ as an absolute contraindication to CMR, especially with 2nd- and 3rd-generation (non-linear) gadolinium agents [10], we adopted this exclusion criteria to ensure patient safety.

Overall, 115 outpatients presenting with a low–intermediate likelihood of O-CAD were included in this study to assess the value of a CCTA plus S-CMR strategy in the diagnostic workup. Pre-test probability of obstructive CAD was calculated according to the new model presented in the 2019 ESC Guidelines for the diagnosis and management of CCS [3], derived from the pooled analysis by Juarez-Orozco et al. [11]. All of these 115 patients underwent CCTA first. Subsequently, those showing intermediate coronary plaques (stenosis 30–70%) underwent S-CMR, including both CAD-RADS 3 and CAD-RADS 2 patients due to compelling clinical history [12]. According to the results of S-CMR, patients were grouped as a positive S-CMR and negative S-CMR group. Revascularization was recommended for symptomatic patients showing ischemia in at least two consecutive left ventricular segments or 6% of the myocardium. ICA and instantaneous wave-free ratio (iFR) were performed in patients with a positive S-CMR, in order to confirm the indication for revascularization. An iFR cut-off value of 0.89 was used [13]. The attending physician conducted the interpretation of the test without any influence. All patients underwent CCTA with a dual-source CTA scanner following scanning protocols conforming to the Society of Cardiac Computed Tomography quality standards [14]. In each coronary artery, coronary atherosclerosis was defined as the presence of any tissue structure >1 mm^2^ either within the coronary artery lumen or adjacent to it that could be discriminated from the surrounding pericardial tissue, epicardial fat, or vessel lumen itself. The severity of the coronary lesions was quantified in multiplanar curved reformatted images by measuring the minimum diameter and reference diameter for all stenoses. All non-evaluable coronary artery segments were censored as positive. No or minimal plaques were defined as <30% stenosis in principal branches of the left or right coronary artery, while intermediate plaques and obstructive plaques were defined as 30–70% and >70% stenosis, respectively. All subjects provided written informed consent, which was approved by the local ethics committee. The study protocol conforms to the ethical guidelines of the 1975 Declaration of Helsinki. All authors had access to all data presented and are responsible for the data integrity.

### 2.1. Endpoint

The primary endpoint was a composite of death from cardiovascular causes, non-fatal myocardial infarction, and unplanned revascularization. As a secondary endpoint, we evaluated recurrent angina despite guideline-directed medical therapy (GDMT).

### 2.2. Follow-Up

The median follow-up was 33.4 months (IQR = 17.7–42.8 months). Information was obtained by clinical visits or telephone interviews, and hospital records of all patients were screened for the occurrence of clinical events to confirm the obtained information.

### 2.3. CCTA Scan Protocol

All CCTA scans were performed using a 128-slice dual-source CTA system (SOMATOM Definition Flash, Siemens Healthineers, Forchheim, Germany). Detector collimation was 2 × 64 × 0.6 mm, using a flying focal spot technique and a gantry rotation time of 280 msec. Scout-based automatic tube current modulation (Care Dose 4D, Siemens Healthcare, Forchheim, Germany) was used with the reference tube current–time product set at 320 mAs per rotation. Oral and/or intravenous beta-blockers or oral ivabradine were administered if necessary, targeting a heart rate of ≤60 bpm. A test bolus scan was performed to determine the transit time. An injection of 15 mL of iodinated contrast medium was followed by a 30 mL saline chaser. The time until the peak opacification in the proximal ascending aorta was measured, and this time plus 2 s for standard protocol and 5 s for high-pitch protocol were considered to represent the transit time of contrast agent. An injection of 65 mL of contrast medium, followed by a 50 mL saline chaser were administered, with bolus tracking using a region of interest (ROI) in the ascending aorta. The scan was automatically triggered when the tracking ROI reached a threshold of 100 Hounsfield units (HUs) above baseline attenuation. In the flash mode (high-pitch spiral mode), prospective ECG triggering was used to obtain a complete data set in a single heart beat starting at 60% of the R–R interval. In the sequential mode (spiral technique), the center of the data acquisition window was set at 70% of the R-R interval. The entire heart was covered in three or four heart beats in a step-and-shoot fashion. Data sets for coronary arteries were reconstructed with a slice thickness of 0.6 mm, an increment of 0.4 mm, a field of view of 180 mm, a medium-soft convolution kernel (B26), and additionally, a sharp convolution kernel (B46) in patients exhibiting coronary calcium. All reconstructed images were transferred to a dedicated workstation (MMWP, Siemens Healthcare, Forchheim, Germany). Axial images, multiplanar reformations, and maximum intensity projections were used to evaluate arteries. Coronary artery segments were classified according to a modified American Heart Association protocol [15]. Segments were evaluated if the luminal diameter met or exceeded 1.5 mm, as judged by two independent observers (N.C. and M.A., each with more than 10 years of CCTA experience). Any discordance interpretation was solved by a third observer (M.M., with more than 8 years of CCTA experience). The radiation dose was reported as dose-length product (DLP) and effective dose (ED). For each patient, the ED was calculated using the formula DLP × 0.014, with a 0.014 conversion factor for chest radiation (in mSv/Gy/cm) [16].

### 2.4. Stress CMR Protocol

All S-CMR scans were performed using a 1.5 T scanner (MAGNETOM ALTEA, Siemens Healthineers, Erlangen, Germany) with an 18-channel anterior body coil. Vasodilatation was induced with dipyridamole infusion at 0.84 mg/kg over 6 min, or with regadenoson 400 mcg bolus over 10 s [17]. After the infusion of the vasodilator agent, a 0.1 mmol/kg bolus of gadolinium-based contrast medium (Dotarem, Guerbet, Paris, France) was injected at a rate of 5.0 mL/s with an injector (Mallinckrodt Optistar Elite, Guerbet S.p.A., Milan, Italy). Stress perfusion imaging was performed using an ECG-triggered saturation-prepared balanced steady-state free precession (bSSFP) sequence with the following typical parameters: repetition time/echo time (TR/TE) = 287/1.2 ms, acceleration factor = 2, field of view = 370 × 314 mm, matrix = 224 × 180, and reconstructed pixel size = 1.7 × 1.7 × 8 mm. A series of five slices (three short-axis views and a 2-chamber and a 4-chamber view) were acquired every other heartbeat. Theophylline was injected intravenously to null the effect of dipyridamole. Ten minutes after the injection of contrast medium, a breath-hold contrast-enhanced T1-weighted inversion recovery gradient-echo sequence was acquired with the same parameters to detect late gadolinium enhancement (LGE). A stress perfusion defect was considered present if it was densest in the endocardium with a transmural gradient across the wall thickness, persisted beyond peak myocardial enhancement for several R-R intervals on electrocardiogram, and conformed to a coronary arterial distribution. Inducible ischemia was defined as the presence of a stress perfusion defect, in the absence of matching LGE, in 2 or more segments. Ischemic scar was defined as the presence of LGE conforming to myocardial infarction in 1 or more segments. All S-CMR scans were interpreted by an experienced reader (N.C. and M.A., each with more than 4 years of S-CMR experience). Any discordance interpretation was solved by a third experienced observer (S.P.).

### 2.5. Statistical Analysis

Continuous variables were expressed as mean ± standard deviation (SD) and categorical variables as frequency with percentage. Normal distribution was assessed using the Shapiro–Wilk test. Follow-up was presented as median and interquartile range (IQR). Differences between patients with and without myocardial ischemia in terms of baseline clinical and S-CMR characteristics were compared using Student’s *t*-test or the Wilcoxon rank sum test for continuous variables and the chi-squared or Fisher’s exact test for categorical variables, as appropriate. Cumulative incidence rates of individual and composite outcomes were estimated using the Kaplan–Meier method and compared with the log-rank test. Statistical analyses were conducted using a commercially available software (SPSS, version 19.0, Chicago, IL, USA). Statistical significance was considered at a two-sided *p*-value below 0.05.

## 3. Results

After the exclusion of patients not fulfilling the inclusion criteria of this study, 115 patients with a suspicion of CCS represent the analyzed population. According to our diagnostic workflow, as a first step, all of these patients underwent CCTA. Based on the CCTA results, patients showing no or only minimal plaques (stenosis < 30%, *n* = 41, 36%) and those with obstructive plaques (stenosis > 70%, *n* = 12, 10%) were excluded from the study. The remaining 62 patients (54%), showing intermediate coronary plaques (30–70% stenosis), underwent S-CMR. After that, according to the results of the S-CMR, patients were grouped as positive S-CMR and negative S-CMR. 

Table 1 shows the demographic and clinical characteristics of the patients. No statistically significant differences in terms of cardiovascular risk factors and associated conditions were observed between the negative S-CMR and positive S-CMR groups, with a mean age of 67.7 ± 9.6 years and a clear preponderance of the male sex (79%), as expected (see Table 1).

Based on the CCTA, 136 intermediate coronary plaques were analyzed: *n* = 27 (20%) were classified as calcified, *n* = 24 (18%) as non-calcified, and *n* = 85 as partially calcified (62%) (see Table 2), without differences between the two groups. Moreover, no statistically significant differences were observed in terms of the coronary plaque distribution between the two groups, even though a trend towards a more proximal coronary location was observed in the positive S-CMR group (see Table 2). The number of segments of coronary vessels that were not evaluable by CTTA (quality score = 1) was low and similar in the negative S-CMR and positive S-CMR groups: 1.9% vs. 3.8%, respectively (*p* = 0.553). The mean cumulative radiation exposure was 3.21 ± 1.29 mSv.

**Figure 2:**
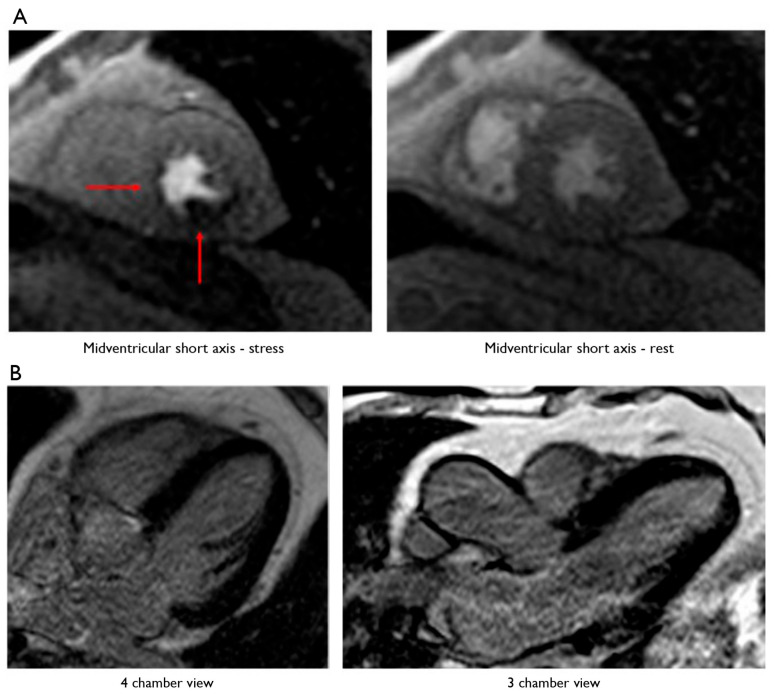
A 65-year-old man was admitted to our department for exertional dyspnea with onset a few months earlier. Both the 12-lead ECG and 2D-echo evaluation did not show any alterations. Considering the low–intermediate risk profile of the patient, CCTA was performed, showing an intermediate plaque on the proximal right coronary artery (RCA) and on the middle circumflex artery (CA). To evaluate their functional significance, a pharmacological stress–rest perfusion and LGE cardiac MRI with regadenoson (0.4 mg IV bolus) were performed (1.5-T MRI system). The left ventricular (LV) short-axis orientation was used for breath hold perfusion imaging, with three sections placed in the basal, midventricular, and apical regions of the LV, using a phased-array surface coil as receiver. In the stress examination, a significant subendocardial perfusion deficit was observed in the basal and middle region of the inferior wall and septum, as well as in the middle posterior wall and postero-lateral papillary muscle. No perfusion deficit was observed in the rest examination. Ten minutes later, using inversion recovery sequences, no LGE was observed. Thus, the patient underwent staged coronary angioplasty with DES placed on the RCA and CA. The patient was discharged uneventful, being free from angina at 3-year follow-up. (**A**) Stress–rest myocardial perfusion MRI in a patient with intermediate plaques on the right and circumflex coronary arteries. The left column shows a short-axis view at the level of the mid-left ventricle during stress (regadenoson), whereas the right column shows a short-axis view at the level of the mid-left ventricle during rest. The red arrows point to a subendocardial perfusion deficit in the middle region of the inferior wall and septum, extending to the basal segment, and in the middle posterior wall and postero-lateral papillary muscle. (**B**) Absence of late gadolinium enhancement (LGE) at cardiac MRI in the myocardial segments, supplied by the right and circumflex coronary arteries. The left column shows a 4-chamber view, and the right column shows a 3-chamber view: no LGE was observed.

Patients showing intermediate coronary plaques at CCTA underwent S-CMR, which resulted in a positive for myocardial perfusion deficit finding in *n* = 17 (27%) and a negative finding in *n* = 45 (73%) patients (Figure 2).

Among the 17 patients with positive S-CMR, 14 underwent ICA plus iFR, and 3 did not due to a known unfavorable coronary anatomy or the patient’s preference of proceeding with medical therapy. The indication for revascularization was confirmed by ICA plus iFR in 13 patients but was not confirmed in 1 patient (iFR = 0.94). According to the results of ICA plus iFR, revascularization was performed through percutaneous coronary intervention (PCI) and drug-eluting stent (DES) implantation in 12 patients and through coronary artery bypass grafting (CABG) in 1 patient, without procedural complications. All patients received GDMT, including high-dose statins, regardless of myocardial revascularization.

### Follow-Up

Clinical follow-up data were available for all patients. Throughout the 1-year follow-up, the primary composite endpoint occurred in one patient (5.9%) belonging to the positive S-CMR group and treated with PCI for unstable angina, and in one patient (2.2%) belonging to the negative S-CMR group, who underwent PCI for persistent refractory exertional dyspnea. The event-free survival curves from the primary composite end-points were not significantly different between the two patient groups (log-rank *p* = 0.485).

**Figure 3:**
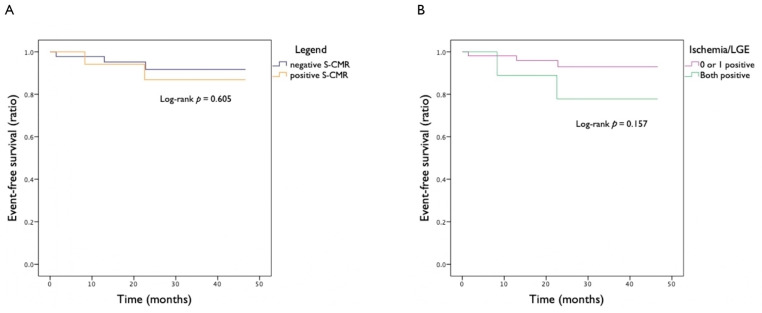
(**A**) Kaplan–Meier analysis of event-free survival from primary composite outcome after a median follow-up of 33.4 months, comparison between positive and negative S-CMR patients. (**B**) Kaplan–Meier analysis of event-free survival from primary composite outcome after a median follow-up of 33.4 months, comparison between patients with positive S-CMR plus presence of ischemic scar and patients with either negative S-CMR and/or absence of ischemic scar.

At the extended follow-up, after a median of 33.4 months (IQR = 17.7–42.8 months), among the positive S-CMR group, the primary composite endpoint occurred in one additional patient (total *n* = 2, 11.8%), who had initially refused ICA after a positive S-CMR test and was thereafter admitted to hospital for an ST-elevation myocardial infarction treated with primary PCI. Among the negative S-CMR group, the primary composite endpoint occurred in two additional patients (total *n* = 3, 6.7%): one treated with PCI for non-ST-elevation myocardial infarction, and another patient who suffered a sudden cardiac death due to a hypertrophic cardiomyopathy associated with a reduced LVEF. The occurrence of the primary composite endpoint was not significantly different between the two groups (log-rank *p* = 0.605), as represented in the Kaplan–Meier graph (Figure 3A), and no statistically significant difference was observed either when comparing patients with positive S-CMR plus a presence of an ischemic scar and patients with either negative S-CMR and/or an absence of an ischemic scar (log-rank *p* = 0.157), as represented in the Kaplan–Meier graph (Figure 3B).

Throughout the extended follow-up, recurrent angina, as a secondary endpoint, was reported by one patient (5.9%) in the positive S-CMR group, showing an unfavorable coronary anatomy that was not suitable for revascularization, whereas no patients (0.0%) in the negative S-CMR group was symptomatic for angina (*p* = 0.274). Throughout the follow-up, no events were observed in patients with no/minimal plaques at CCTA.

## 4. Discussion

In this study, we found that a CCTA plus S-CMR strategy works very well in clinical practice. First, we can safely avoid unnecessary further examination in a significant proportion of CCS patients showing no or only minimal plaques at CCTA, confirming its high negative predictive value [18]. More importantly, for CCS patients with intermediate coronary plaques at CCTA, the strategy of adding S-CMR appears to be effective, leading us to refine the selection of patients needing ICA and coronary revascularization, who in our study population were as many as a quarter of the patients. Finally, in all CCS patients, GDMT may have contributed to freedom from angina, regardless of coronary revascularization. 

Current guidelines on the management of patients with CCS recommend the use of CCTA for patients with low–intermediate risk and S-CMR in different diagnostic pathways [3,4]. The MR INFORM trial [9] demonstrated that S-CMR is non-inferior to ICA with measurement of FFR in guiding the management of patients with stable CAD with respect to major adverse cardiac events. Thus, we believe that both CCTA and S-CMR should be implemented in clinical practice to improve the diagnostic and therapeutic management of CCS patients. 

Currently, there is significant global in the cardiology community about the benefits of revascularization in patients with angina, clinically significant myocardial ischemia, or hemodynamically relevant CAD [19,20,21,22]. Several factors may explain the different results in previous trials involving patients with stable CAD. In previous trials in which various revascularization methods were compared with the best available medical therapy, patient enrollment was primarily based on angiographic findings, with or without noninvasive documentation of ischemia. It is likely that a sizable proportion of the patients had only limited ischemia. Even in the COURAGE (Clinical Outcomes Utilizing Revascularization and Aggressive Drug Evaluation) trial, in which noninvasive testing was performed in 85% of the patients [19], only less than one-third of the patients had more than 10% ischemia on myocardial perfusion imaging [23]. Not surprisingly, in daily clinical practice, less than half of the patients undergo noninvasive stress testing before elective PCI [5,24]. In the FAME 2 trial [21], FFR-guided PCI with DES plus the best available medical therapy, as compared with the best available medical therapy alone, resulted in significantly improved clinical outcomes, driven by an increase by a factor of 8 in the need for urgent revascularization in the medical therapy alone group. The recent International Study of Comparative Health Effectiveness with Medical and Invasive Approaches (ISCHEMIA) did not demonstrate any beneficial effect of revascularization in patients with a moderate-to-severe burden of myocardial ischemia [25], and this picture did not change at the extended follow-up [26]. In addition, the REVIVED-BCIS2 trial [27] failed to demonstrate a major efficacy of PCI in comparison to GDMT in patients with LV systolic dysfunction, multivessel disease, and myocardial viability. Thus, worldwide in the cardiology community, the beneficial effect of coronary revascularization for CCS remains a matter of debate. Importantly, taking into account the results of the ISCHEMIA, ISCHEMIA EXTENDED, and REVIVED-BISC2 trials [25,26,27], one can hypothesize the incremental beneficial effect of contemporary GDMT, as well as the potential lack of effectiveness of revascularization in the majority of CCS patients. Certainly, the management of CCS patients in the real world remains at the discretion of the attending physician. In this scenario, the adoption of a CCTA plus S-CMR strategy appears useful for an appropriate selection of CCS patients needing functional coronary evaluation and, consequently, for an appropriate selection of CCS patients needing coronary revascularization. The results of our study show that a CCTA plus S-CMR strategy is safe in clinical practice, since the outcome was not different between patients who did or did not undergo ICA according to the results of S-CMR. Furthermore, in our study, the incidence of outcome events at 3 years was lower than expected on the basis of data from the ISCHEMIA trial [25] (which enrolled patients with documented moderate-to-severe myocardial ischemia and a severe burden of CAD). On the other hand, a lower incidence of events in our small study population is in agreement with the results of larger registries, highlighting the prognostic value of clinical routine CMR for this indication [28,29]. Obviously, adherence to the best available medical therapy, including statins and lifestyle changes, was high in our study, contributing to the lower rate of events, as well as to the freedom from angina in the vast majority of our patients. In addition, based on the results of our study, we can safely avoid unnecessary further examination in CCS patients showing no/minimal plaques at CCTA. The present results are in direct accordance with the findings of a large study, the CONFIRM registry, where patients without evident CAD by CCTA who were followed for ≥4 years had an extremely favorable prognosis, with a 0.22% annualized death rate [30]. Such a low mortality rate validates the favorable prognosis and emphasizes the clinical value of CCTA for the identification of individuals in whom no further additional testing and/or therapy is necessary or indicated. With the incremental use of CCTA, it became evident that in comparison to the absence of CAD, non-obstructive CAD negatively affects the prognosis (in particular its severity and extent), albeit to a smaller extent compared to obstructive CAD. Therefore, it is necessary to consider non-obstructive CAD within the cardiovascular risk continuum. Currently, guidelines do not provide a specific therapy for non-obstructive CAD [3]. However, it is reasonable to identify this condition as a ‘risk modifier’ and therefore as a further indicator of increased cardiovascular risk [31], in order to implement specific measures that are able to reduce the risk of events, as suggested by recent clinical trials [32,33]. In the current era of CCTA, the need to look not only at single plaque features or stenosis, but also at the extent of CAD as a better predictor of the patient outcome is increasingly clear [25,26].

### Study Limitations

This study was a single-center, observational registry with several limitations. First, the small sample size implied that no firm conclusions can be drawn, and caution is needed in the interpretation of these results. In addition, we cannot determine whether the knowledge of coronary anatomy from CCTA could have influenced the S-CMR interpretation. Moreover, a bias towards revascularization in the positive S-CMR group of patients cannot be fully ruled out, since coronary revascularization after ICA plus iFR was left at the discretion of the interventional cardiologist. Undoubtably, the additional prognostic value of the presence of an ischemic scar in our study is not fully appreciated due to the small sample size. Additionally, the mean follow-up period of 3 years may mask some possible longer-term differences between the two groups of patients. Moreover, the appropriateness of extending our findings to other centers relies on the comparability of the clinical setting in terms of the diagnostic practices, available technology, cost-accounting approach, and management of the care system. The results of S-CMR cannot be extended to other methods for testing the presence of myocardial ischemia or the functional significance of a coronary artery stenosis because of their differences in diagnostic performance compared with S-CMR. Finally, while the CCTA plus S-CMR strategy may be cost-saving, we did not perform a cost analysis of the integration of S-CMR in the workflow for suspicion of CAD at our tertiary center. In accordance with a sustainable imaging cost policy for patients with suspicion of CCS, the FFR-CCTA and the fusion/hybrid imaging is not available in our institution.

## 5. Conclusions

Our study suggests that the adoption of a CCTA plus S-CMR strategy for the evaluation of patients with suspicion of CCS at low–intermediate risk may help refine the selection of patients with intermediate coronary plaques at CCTA needing coronary revascularization. The outcome was not different between patients who did or did not undergo ICA according to the results of S-CMR, highlighting the efficacy of the CCTA plus S-CMR strategy in this setting. In all CCS patients, GDMT may have contributed to freedom from angina, regardless of coronary revascularization.

## Figures and Tables

**Table 1:** Demographic and clinical characteristics and medical therapy of patients.

Demographic and Clinical Characteristics	Total (*N* = 62)	Negative S-CMR (*N* = 45)	Positive S-CMR (*N* = 17)	*p*
Age—year	67.7 ± 9.6	67.1 ± 9.8	69.3 ± 9.0	0.405
Sex				0.746
- Male	49 (79.0%)	35 (77.8%)	14 (82.4%)	
- Female	13 (21.0%)	10 (22.2%)	3 (17.6%)	
BMI	27.2 ± 3.2	26.9 ± 2.6	27.4 ± 3.6	0.733
Hypertension	43 (69.4%)	30 (66.7%)	13 (76.5%)	0.545
Diabetes mellitus	15 (24.2%)	9 (20.0%)	6 (35.3%)	0.197
Dyslipidemia	46 (74.2%)	34 (75.6%)	12 (70.6%)	0.745
Current or previous smoker	31 (50.0%)	22 (48.9%)	9 (52.9%)	0.779
Positive family Hx for CAD	16 (25.8%)	10 (22.2%)	6 (35.3%)	0.328
Pre-test probability of O-CAD	29.0%	28.4%	32.0%	
CKD	4 (6.5%)	3 (6.7%)	1 (5.9%)	0.999
COPD	1 (1.6%)	1 (2.2%)	0 (0.0%)	0.999
PAD	6 (9.7%)	4 (8.9%)	2 (11.8%)	0.999
History of stroke	1 (1.6%)	0 (0.0%)	1 (5.9%)	0.288
LVEF < 40%	1 (1.6%)	1 (2.2%)	0 (0.0%)	0.999
**Medical Therapy**				
Aspirin	47 (75.8%)	33 (73.3%)	14 (82.3%)	0.528
Beta-blockers	31 (50.0%)	24 (53.3%)	7 (41.2%)	0.570
ACE inhibitors/ARBs	37 (59.7%)	26 (57.8%)	11 (64.7%)	0.773
Statins	51 (82.3%)	37 (82.2%)	14 (82.3%)	0.999
Nitrates	3 (4.8%)	1 (2.2%)	2 (11.8%)	0.179
Calcium channel blockers	22 (35.5%)	13 (28.9%)	9 (52.9%)	0.135

Values reported as mean ± standard deviation or frequency and percentage (%). ACE, angiotensin-converting enzyme; ARBs, angiotensin receptor blockers; BMI, body mass index; CAD, coronary artery disease; CKD, chronic kidney disease; COPD, chronic obstructive pulmonary disease; Hx, history; LVEF, left ventricular ejection fraction; O-CAD, obstructive coronary artery disease; PAD, peripheral artery disease; S-CMR, stress cardiac magnetic resonance.

**Table 2:** CCTA plaques’ characteristics.

Analysis by Patients	Total (*N* = 62)	Negative S-CMR (*N* = 45)	Positive S-CMR (*N* = 17)	*p*
**Coronary arteries:**				
Left main	2 (3.2%)	2 (4.4%)	0 (0.0%)	0.820
LAD	43 (69.3%)	31 (68.9%)	12 (70.6%)	
Circumflex	26 (41.9%)	18 (40.0%)	8 (47.1%)	
Right coronary	26 (41.9%)	18 (40.0%)	8 (47.1%)	
**Analysis by segments**				
Proximal part	31 (50.0%)	20 (44.4%)	11 (64.7%)	0.684
Middle part	28 (45.2%)	18 (40.0%)	8 (47.1%)	
Distal part	20 (32.3%)	15 (33.3%)	5 (29.4%)	
**Analysis by plaques**	**Total** **(*N* = 136)**	**Negative S-CMR** **(*N* = 107)**	**Positive S-CMR** **(*N* = 29)**	
Calcified	27 (19.9%)	22 (20.6%)	5 (17.3%)	0.578
Non-calcified	24 (17.6%)	17 (15.9%)	7 (24.1%)	
Partially calcified	85 (62.5%)	68 (63.5%)	17 (58.6%)	

Values reported as frequency and percentage (%). LAD, left anterior descending; S-CMR, stress cardiac magnetic resonance.

## Data Availability

The raw data supporting the conclusions of this article will be made available by the authors on request.

## Abbreviations

bSSFP	balanced steady-state free precession
CABG	coronary artery bypass grafting
CAD	coronary artery disease
CCS	chronic coronary syndrome
CCTA	coronary computed tomography angiography
DES	drug-eluting stent
DLP	dose-length product
ED	effective dose
ESC	European Society of Cardiology
FFR	fractional flow reserve
GDMT	guideline-directed medical therapy
HUs	Hounsfield units
ICA	invasive coronary angiography
iFR	instantaneous wave-free ratio
IQR	interquartile range
LGE	late gadolinium enhancement
LV	left ventricle
O-CAD	obstructive coronary artery disease
PCI	percutaneous coronary intervention
ROI	region of interest
S-CMR	stress cardiac magnetic resonance imaging
SD	standard deviation
TR/TE	repetition time/echo time
